# Spatio-temporal stochastic differential equations for crime incidence modeling

**DOI:** 10.1007/s00477-022-02369-x

**Published:** 2023-01-04

**Authors:** Julia Calatayud, Marc Jornet, Jorge Mateu

**Affiliations:** 1 Departament de Matemàtiques, Universitat Jaume I, 12071 Castellón, Spain; 2 Departament de Matemàtiques, Universitat de València, 46100 Burjassot, Spain

**Keywords:** Space-time crime data, Differential equations, Trend time series, Geometric Brownian motion stochastic process, District-level correlations, 60H10, 62M10, 62M30, 62P25

## Abstract

We propose a methodology for the quantitative fitting and forecasting of real spatio-temporal crime data, based on stochastic differential equations. The analysis is focused on the city of Valencia, Spain, for which 90247 robberies and thefts with their latitude-longitude positions are available for a span of eleven years (2010–2020) from records of the 112-emergency phone. The incidents are placed in the 26 zip codes of the city (46001–46026), and monthly time series of crime are built for each of the zip codes. Their annual-trend components are modeled by Itô diffusion, with jointly correlated noises to account for district-level relations. In practice, this study may help simulate spatio-temporal situations and identify risky areas and periods from present and past data.

## Introduction

Mathematical criminology is a current field of research which uses mathematical methods for understanding and predicting the incidence of crime. A proper mathematical analysis may help make the best use of the existing, limited public resources.

This paper is placed in the context of differential equation models for crime evolution. Differential equations relate characteristics and their rates of change at different space and/or time positions, which is particularly useful for describing growth or decreases of incidences, fluxes between segments, diffusion, etc. Two types of differential equations have been applied in mathematical criminology: partial differential equations and ordinary differential equations.

Partial differential equations often aim at identifying space-time clusters of crime, referred to as hotspots (Berestycki et al. 2013; Gu et al. 2017; Kolokolnikov et al. 2014; Manásevich et al. 2013; Rodriguez and Bertozzi 2010; Short et al. 2010a, b; Tse and Ward 2015). Dynamical systems theory helps understand the changes in these hotspots, for example under police intervention. On the other hand, ordinary differential equations study fluxes of people between compartments by social interaction (for instance, pressure or persuasion to become an offender) (Abbas et al. 2017; González-Parra et al. 2018; McMillon et al. 2014; Misra 2014; Srivastav et al. 2019, 2020). The problem with these types of models is that they are not usually amenable to fitting real crime data, due to the complexities involved in the formulations and the lack of records.

In the literature, we found only four differential equation-based contributions that fit actual crime time-series data. In what follows, we comment their methodology and limitations, to better motivate our investigation.

In paper (Lacey and Tsardakas 2016), the authors studied serious (such as burglaries or violent crime) and minor (e.g. shoplifting) incidents in Manchester. Based on ideas from diffusion partial differential equations, they proposed a three-dimensional system of ordinary differential equations, taking into account attractiveness (indicator of how likely it is for a criminal to act at a specific time). Monthly data, with no spatial segmentation, were fitted by least-squares optimization, but a big challenge was the fact that the parameters were not identifiable and the inverse problem did not show uniqueness. The paper also suggested the incorporation of Brownian-type stochastic components to capture fluctuations, but the stochastic model was not calibrated. All these issues were discussed in their article.

In paper (Jane White et al. 2021), the authors proposed a two-dimensional system of ordinary differential equations to study crime evolution, based on people fluxes (self-initiation to crime, peer pressure, ceased criminality). The model was applied to yearly data from South-Africa, in the period 2005–2016. The region was halved into high- and low-conflicting areas, according to the threshold of 1 murder for every 1000 inhabitants in 2016. The parameters were estimated by Bayesian inference, albeit the results reported were deterministic. The training period corresponded to the years 2005–2009. Increasing crime patterns were observed. Since data were aggregated on an annual basis, noisy patterns did not arise and stochastic equations were not employed.

In the UCLA report (Cao et al. 2013), the authors considered daily burglary data from Los Angeles (California) and Houston (Texas) for the periods 2005–2013 and 2009–2013, respectively. Due to the noisy features of the two time series, the authors extracted the trend component of the series. Each trend time series was modeled by a two-dimensional Lotka-Volterra stochastic differential equation, with independent Wiener processes. Historical data were fitted and a past missed period (near the end of 2007 and the beginning of 2008) was reproduced. It is remarked that Los Angeles and Houston were not modeled together seeking possible interactions. Rather, the parameters corresponding to each city were estimated independently. A least-squares fitting procedure was employed to calibrate the parameters of the deterministic part of the Lotka-Volterra model, by matching the predator function with the trend crime data of the city. The noises’ intensities were then fixed by likelihood maximization. For numerical computations, a nonstandard Euler-Maruyama scheme was used.

The fourth contribution, article (Calatayud et al. 2023a), was recently published by the authors of the present paper. We considered crime data in the city of Valencia, Spain, notified to the 112-emergency phone for the years 2010–2020. The dataset distinguished between aggression (a theft after hitting a person), stealing (a smooth theft with no force used), woman alarm (a theft to a woman with violence), and others (thefts or robberies that cannot be considered within the previous three groups). The interest relied on the modeling of the three monthly time series corresponding to the events of aggression, stealing and woman alarm. Each time series was decomposed into trend and seasonality. The former was modeled by geometric Brownian motion and the latter was fitted by randomly perturbed sine-cosine waves. Also, the interaction between two crime events, such as aggression and stealing, was analyzed by correlating two Brownian motions. The numerical results showed that the models, albeit simple, matched the data well. Multidimensional correlations, beyond two Brownian motions, or spatial effects were not studied. A comparison with mechanistic models was made and research lines were proposed.

Motivated by the analysis from Calatayud et al. (2023a) and the fifth research line suggested in its discussion section, in the present paper we investigate the use of stochastic differential equations for the spatio-temporal modeling of real crime data series. Again, the study is centered on the records for the city of Valencia, Spain, along the years 2010–2020. We pick ideas from the interesting report (Cao et al. 2013) and our recent contribution (Calatayud et al. 2023a). Likewise, we use trend components to apply Itô diffusion. However, we are interested in the existing spatial correlations of crime in Valencia. We work with latitude-longitude positions and zip codes, and correlate all noises to incorporate spatial effects. Spatial interactions were not analyzed in Cao et al. (2013), Calatayud et al. (2023a). Based upon (Calatayud et al. 2023a), we conduct the investigation with the geometric Brownian motion stochastic process, which is a stochastic differential equation used in quantitative finance (Lamberton and Lapeyre 2011). The adopted approach is simple and does not pose difficulties for parameter calibrations or computations.

It is a good point to introduce, interpret and motivate the key concept of correlation for stochastic processes. Given two stochastic processes $u_t$ and $v_t$, their correlation is

$$\begin{aligned} \text{corr}[u_t,v_t]=\frac{{\mathbb {E}}[(u_t-{\mathbb {E}}[u_t])(v_t-{\mathbb {E}}[v_t])]}{\sqrt{{\mathbb {V}}[u_t]}\sqrt{{\mathbb {V}}[v_t]}}\in [-1,1], \end{aligned}$$

where ${\mathbb {E}}$ and ${\mathbb {V}}$ denote the expectation and the variance operators, respectively (Casella and Berger 2002, Section 4.5). Correlation measures how similar the behaviors of the two processes around their mean values are, on a linear basis. Essentially, it is useful for identifying common patterns in unexpected changes of processes. In practice, if a certain process starts deviating from its expected path strongly, then practitioners should put their attention on the correlated processes and reallocate resources. In this article, our processes are related to trends of crime time series in different spatial districts and involve spatial reallocation of police over time.

The structure of the paper is as follows. In Sect. 2, we present our case study and we describe the methods to process the available data and to model the time series of crime. In Sect. 3, the results obtained are explained and accompanied with plots and tables: spatial partition of the data, correlations between patches, calibrations of model parameters, fit of past data and forecasts. Finally, Sect. 4 is devoted to the discussion of the main aspects of the paper, a comparison with the literature, and limitations and possible extensions.

## Methods

In this section, we detail the treatment of the crime data and the spatio-temporal modeling of the time series with stochastic differential equations.

### Data processing

**Fig. 1:**
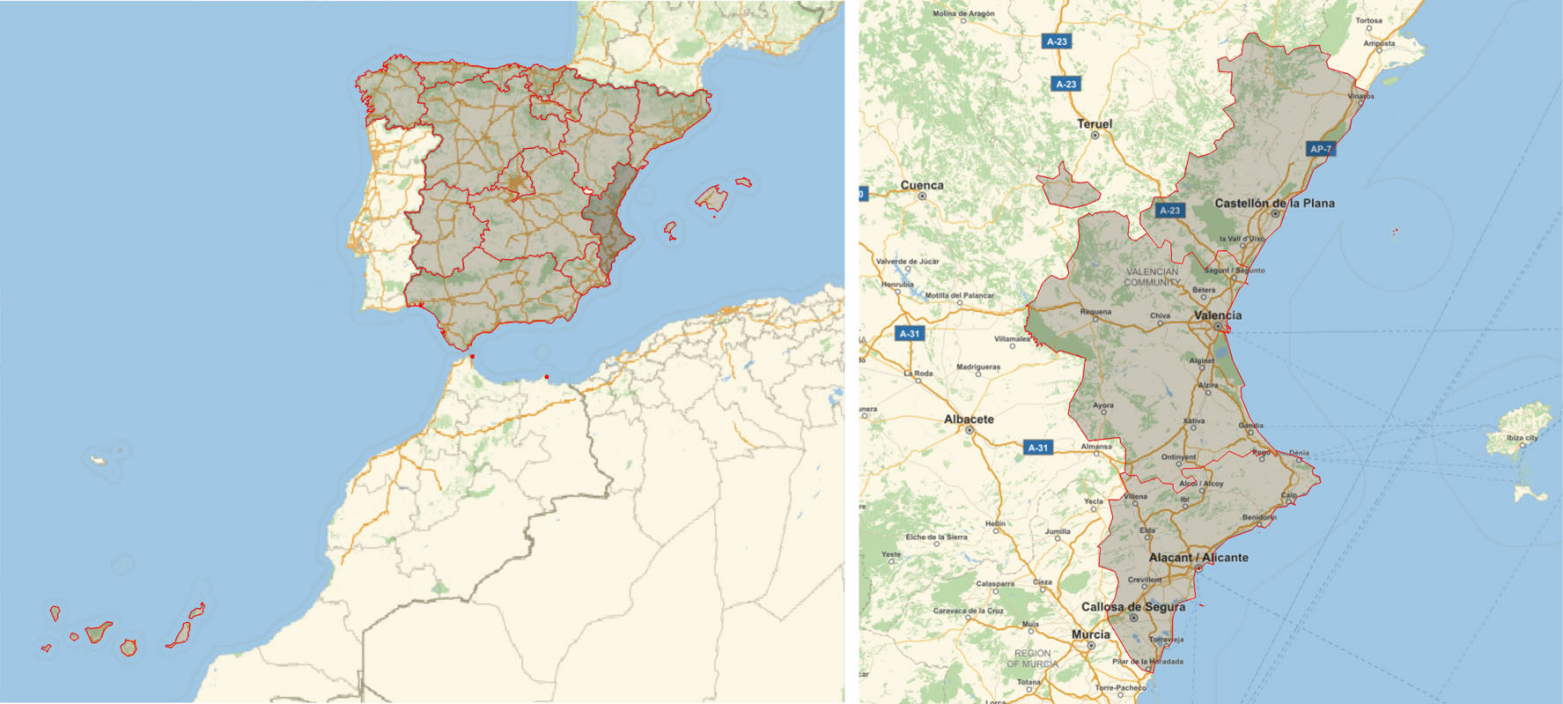
Location of the Valencian region among the autonomous communities of Spain (first map), and the three provinces of the Valencian region with the city of Valencia in the middle (second map). The borders of the autonomous communities and the three provinces are marked in red. Source: Mathematica^®^ (Wolfram Research 2020), built-in function *GeoGraphics*

This study is focused on the city of Valencia, Spain. Located in the Mediterranean coast, it is the capital of the Valencian region and has a population of around 800000 inhabitants (ranked third in Spain). Figure 1 displays the locations of the Valencian region within Spain (first panel) and of the city of Valencia (second panel).

Our dataset contains daily information about criminal events in Valencia, from January 2010 to December 2020. There are 90247 incidents communicated to the 112-emergency phone and consequently verified, which correspond to violent or smooth robberies or thefts in the streets. (We note that the types of crime will not be distinguished here.) Each incident is located in the city by its latitude and longitude position. For modeling purposes, we treat the data as follows: we use absolute counts by aggregating on a monthly basis, and we assign a zip code (a patch) to each latitude-longitude location (among the 26 codes existing in Valencia, from 46001 until 46026). This procedure gives 26 time series with $12\text { months}\times 11\text { years}=132$ counts each, reflecting monthly criminal events at each zip code for eleven years. As differential equations constitute our main tool for spatial modeling, the built time series need to have sufficient records; this is the reason of working with months and zip codes instead of days, streets or spatial points.

The assignment of zip codes to each *xy*-position is performed as follows. The official web page at Las Provincias (https://www.lasprovincias.es/valencia-ciudad/listado-codigos-postales-valencia-calles-20210205144912-nt.html) provides streets of Valencia (nearly 4000) with their zip codes. We select around 20 streets for each code and look up their coordinates at Google Maps (Google 2022). We then have representatives of latitude-longitude locations at each zip code. For the 90247 crime events and their latitude and longitude, we compute the Euclidean distances with respect to the representatives and select the zip code with minimum distance. In this manner, we have a partition of the 90247 events into 26 patches. For modeling, we focus on the 26 time series that these data generate.

### Time-series modeling

The 26 time series are highly noisy, due to the abrupt monthly variability of the number of crime incidents. As suggested in the UCLA report (Cao et al. 2013), the extraction of a trend component permits smoothing out the original time series and modeling the resulting part with Itô diffusion. The trend is computed by a moving average on an annual basis (the average per 12 months, due to observed seasonality), which gives rise to a total length of 121. With stochastic differential equations of Itô type (Allen 2007; Evans 2012; Mao 2007), which can be thought of as a type of differential equation with randomness built in, we will try to capture the yearly average incidence of crime in Valencia. Here, by “capturing” we mean constructing expected values and probabilistic regions, as well as generating realizations that mimic the fluctuating patterns of the trends. For past history, an optimal path may be generated for fitting the trends quantitatively. For forecasting, however, pointwise quantitative predictions cannot be expected with randomly fluctuating dynamics, and mean values and credible regions are used after a training period.

The stochastic differential equation that will be employed here is a simple one, based on the financial literature (Lamberton and Lapeyre 2011): the geometric Brownian motion, which leads to the Black-Scholes partial differential equation for options pricing. Our trend time series do not have any financial interpretation, but their fluctuations and dynamics are visually similar to those of stock prices. Further, as will be seen, the geometric Brownian motion is suited to incorporate spatial dependencies easily, in contrast to more complex mathematical models based on fluxes between patches (which suffer from the curse of dimensionality or the absence of parameter identifiability) or partial differential equations (which are difficult to calibrate from data) (van den Driessche 2008; Wu 2008). In terms of time-series modeling, spatial dependencies between patches in the same jurisdiction are somewhat similar to assets’ dependencies between companies in the same financial market.

Let us revisit the construction of the geometric Brownian motion, with strong emphasis on our context of crime modeling.

First, it is necessary to begin with the deterministic model. Given one of the 26 zip codes, we start with the absolute number of criminals that commit robberies or thefts there, modeled by a function of time $\{c_t\}_{t\ge 0}$. The simplest ordinary differential equation is given by the exponential model,

2.1 $$\begin{aligned} c_t'=\mu c_t, \end{aligned}$$

where the prime denotes the derivative with respect to time, $\mathrm{d}/\mathrm{d}t$, and $\mu \in {\mathbb {R}}$. This parameter $\mu$ controls the inflow rate from susceptibility to criminality by social pressure, imitation or persuasion (Burgess and Akers 1966; Esiri 2016; Harkins et al. 2017), $\mu _{\text {in}}>0$, and the outflow rate from criminality to susceptibility by cessation, $\mu _{\text {out}}>0$, so that $\mu =\mu _{\text {in}}-\mu _{\text {out}}$:

$$\begin{aligned} c_{t+\mathrm{d}t}=c_t+\underbrace{(\mu _{\text {in}}\cdot \mathrm{d}t)\cdot c_t}_{\text {inflow}}-\underbrace{(\mu _{\text {out}}\cdot \mathrm{d}t)\cdot c_t}_{\text {outflow}}. \end{aligned}$$

It is similar to a birth-death environment, largely studied in population ecology (Turchin 2001). Another interpretation for the criminological exponential model is the following. Let

$$\begin{aligned} {\mathcal {T}}_1= {}&\text {time a criminal needs to convince or stimulate some susceptible} \\ {}&\text {person, since the beginning or again after a previous persuasion} \\ {}&\sim \mathrm{Exp}(\mu _{\text {in}}); \\ {\mathcal {T}}_2= {}&\text {time of criminality stay until reintegration in society} \\ {}&\sim \mathrm{Exp}(\mu _{\text {out}}); \end{aligned}$$

$$\begin{aligned} c_{t|1,s}= {}&\text {number of criminals at } t \text { whose last influence to a susceptible was} \\ {}&\text {made at instant } s, s\le t; \end{aligned}$$

$$\begin{aligned} c_{t|2,s}= {}&\text {number of criminals at } t \text { whose last incorporation into criminality} \\ {}&\text {occurred at instant } s, s\le t. \end{aligned}$$

The set $\{s:\, s\le t,\; c_{t|i,s}\ne 0\}$ is finite, $i\in \{1,2\}$, because there is a finite discrete number of offenders. Criminality evolves as

$$\begin{aligned} c_{t+\mathrm{d}t}= {}&c_t + \sum _{s\le t} \mathrm{Pr}[{\mathcal {T}}_1\le t-s+\mathrm{d}t|{\mathcal {T}}_1>t-s]c_{t|1,s} \\ - {}&\sum _{s\le t} \mathrm{Pr}[{\mathcal {T}}_2\le t-s+\mathrm{d}t|{\mathcal {T}}_2>t-s]c_{t|2,s}, \end{aligned}$$

where $\mathrm{d}t>0$ and $\mathrm{Pr}[\star | \star ]$ is the conditional probability, here acting as a proportion. Taking into account the hazard function (the instantaneous-relative-risk function) of the exponential distribution (Evans et al. 2000, page 13),

$$\begin{aligned}{} & {} \frac{\mathrm{Pr}[{\mathcal {T}}_1\le t-s+\mathrm{d}t|{\mathcal {T}}_1>t-s]}{\mathrm{d}t} {\mathop {\longrightarrow }\limits ^{\mathrm{d}t\rightarrow 0}} \mu _{\text {in}},\\{} & {} \quad \frac{\mathrm{Pr}[{\mathcal {T}}_2\le t-s+\mathrm{d}t|{\mathcal {T}}_2>t-s]}{\mathrm{d}t}{\mathop {\longrightarrow }\limits ^{\mathrm{d}t\rightarrow 0}} \mu _{\text {out}}, \end{aligned}$$

the exponential model (2.1) is obtained. In conclusion, the coefficient $\mu$ is a balance between the relative risks of criminal influence and reintegration.

Now, it does not matter whether we work with number of criminal acts or with number of criminal persons, as ones assumes a proportional relationship in terms of average number of crimes committed per criminal: $\text {incidents}=\alpha \times \text {criminals}$, $\alpha >0$ (Jane White et al. 2021). With mathematical functions, let $\{y_t\}_{t\ge 0}$ be the modeled number of robberies and thefts at the zip code. Then $y_t=\alpha c_t$ is the mentioned proportional relationship between incidents and criminals, and

2.2 $$\begin{aligned} y_t'=\alpha c_t'=\alpha \mu c_t=\mu y_t, \end{aligned}$$

by (2.1). The trend of the time series is modeled by a function of time $\{x_t\}_{t\ge 0}$, defined as the annual moving average

$$\begin{aligned} x_t=\frac{y_t+y_{t+1}+\ldots +y_{t+11}}{12}. \end{aligned}$$

Then, by linearity, we derive the same ordinary differential equation as in (2.1) and (2.2),

2.3 $$\begin{aligned} x_t'=\mu x_t. \end{aligned}$$

This is the deterministic mathematical model for the trend evolution. Although simple, it has required an in-depth analysis.

As there are random factors that may affect the risk of criminality along time, the parameter $\mu$ in (2.3) is perturbed by a Gaussian white noise process with intensity (magnitude) $\sigma >0$:

$$\begin{aligned} \mu \leftarrow \mu +\sigma B_t'. \end{aligned}$$

The Gaussian noise $B_t'$ is idealized (a Schwartz distribution or generalized process), uncorrelated with infinite variance and zero mean, and it is viewed as the formal derivative of a standard Brownian motion, or Wiener process, $B_t$. Brownian motion is a Gaussian process with the properties of zero mean, covariance given by the minimum of the two time instants, and independent increments; its trajectories are continuous but nowhere differentiable or monotone. Then, model (2.3) for the trend becomes stochastic:

$$\begin{aligned} x_t'=\mu x_t+\sigma x_t B_t'. \end{aligned}$$

The noise is proportional to the incidence of crime; then higher variability occurs when crime presents higher rates. In differential notation, the model takes the form of an Itô equation:

2.4 $$\begin{aligned} \mathrm{d}x_t=\mu x_t \,\mathrm{d}t+\sigma x_t\,\mathrm{d}B_t. \end{aligned}$$

It indicates that the infinitesimal growth rate, $(x_{t+\mathrm{d}t}-x_t)/x_t$, has a normal distribution (i.e. the maximum-entropy distribution (Dorini and Sampaio 2012)) with mean value $\mu \,\mathrm{d}t$ and variance $\sigma ^2\,\mathrm{d}t$, given $\mathrm{d}t>0$; and besides, the infinitesimal growth rates are independent^1^. Rigorously, the differential model is interpreted in integral form under the theory of Itô calculus. Now the solution is a stochastic process $x_t$, called geometric Brownian motion. By Itô lemma^2^, which extends the standard chain rule theorem for non-differentiable processes, the solution to (2.4) is given by

2.5 $$\begin{aligned} x_t=x_0 \mathrm{e}^{(\mu -\frac{1}{2} \sigma ^2)t+\sigma B_t}, \end{aligned}$$

where $x_0>0$ is the initial, deterministic state. The expected value of $x_t$ coincides with the solution to the deterministic model (2.3), $x_0\mathrm{e}^{\mu t}$. The stochastic solution (2.5) entails random variability and is qualitatively closer to data. Its trajectories are positive and continuous but nowhere differentiable or monotone. A probabilistic interval for $x_t$ at level $1-\alpha$ is given by

$$\begin{aligned} \left[ x_0 \mathrm{e}^{(\mu -\frac{1}{2} \sigma ^2)t-\sigma \cdot \sqrt{t}\cdot z_{\alpha /2}},x_0 \mathrm{e}^{(\mu -\frac{1}{2} \sigma ^2)t+\sigma \cdot \sqrt{t}\cdot z_{1-\alpha /2}}\right] , \end{aligned}$$

where *z* stands for the quantile function of a standard normal distribution.

This stochastic model (2.4) fits a single trend time series. But it would be advisable to incorporate certain spatial structure, because crime incidence might present correlations for different zip codes. The idea is that, although each zip code has its own geometric Brownian motion for its trend time series, the Brownian motions are correlated. Indeed, the random factors that may affect the risk of criminality are not entirely independent among patches. Mathematically, given *n* trend time series (in our case, $n=26$) modeled as

2.6 $$\begin{aligned} \mathrm{d}x_{1,t}= {}&\mu _1 x_{1,t} \,\mathrm{d}t+\sigma _1 x_{1,t}\,\mathrm{d}B_{1,t}, \nonumber \\ {}&\ldots \nonumber \\ \mathrm{d}x_{n,t}= {}&\mu _n x_{n,t} \,\mathrm{d}t+\sigma _n x_{n,t}\,\mathrm{d}B_{n,t}, \end{aligned}$$

with stochastic solutions

2.7 $$\begin{aligned} x_{1,t}= {}&x_{1,0} \mathrm{e}^{(\mu _1-\frac{1}{2} \sigma _1^2)t+\sigma _1 B_{1,t}}, \nonumber \\ {}&\ldots \nonumber \\ x_{n,t}= {}&x_{n,0} \mathrm{e}^{(\mu _n-\frac{1}{2} \sigma _n^2)t+\sigma _n B_{n,t}}, \end{aligned}$$

respectively, the Brownian motions satisfy

2.8 $$\begin{aligned} \mathrm{corr}[B_{i,t},B_{j,t}]=\rho _{ij} \in [-1,1], \end{aligned}$$

for all $t\ge 0$ and labels $i,j\in \{1,2,\ldots ,n\}$. The construction of this set of Brownian motions is not difficult, by using the properties of covariance matrices (Xiu 2010, Section 4.1.1): given the correlation matrix $\mathrm{A}=(\rho _{ij})_{i,j}$ and given auxiliary independent Brownian motions ${\tilde{B}}_{1,t}=B_{1,t}$, ${\tilde{B}}_{2,t},\ldots ,{\tilde{B}}_{n,t}$, just define

$$\begin{aligned} \begin{pmatrix} B_{1,t} \\ \vdots \\ B_{n,t} \end{pmatrix} = \mathrm{L} \begin{pmatrix} {\tilde{B}}_{1,t} \\ \vdots \\ {\tilde{B}}_{n,t} \end{pmatrix}, \end{aligned}$$

where $\mathrm{L}$ is a lower-triangular matrix and $\mathrm{A}=\mathrm{L}\mathrm{L}^\top$ is the Cholesky decomposition of the symmetric and positive definite matrix $\mathrm{A}$. For example, for a pair of regions, we have a Brownian process $B_{1,t}$ and we define

$$\begin{aligned} B_{2,t}=\rho B_{1,t}+\sqrt{1-\rho ^2}\,{\tilde{B}}_{2,t}, \end{aligned}$$

where ${\tilde{B}}_{2,t}$ is an auxiliary Brownian motion that is independent of $B_{1,t}$; then $\mathrm{corr}[B_{1,t},B_{2,t}]=\rho$.

For a better understanding of the role of $\rho _{ij}$ beyond (2.8), it is interesting to observe in (2.6) that

$$\begin{aligned} \mathrm{cov}[\mathrm{d}x_{i,t},\mathrm{d}x_{j,t}|x_{i,t},x_{j,t}] = {}&\sigma _i\sigma _jx_{i,t}x_{j,t} \mathrm{cov}[\mathrm{d}B_{i,t},\mathrm{d}B_{j,t}|x_{i,t},x_{j,t}]\\&=\sigma _i\sigma _jx_{i,t}x_{j,t} \mathrm{cov}[\mathrm{d}B_{i,t},\mathrm{d}B_{j,t}] \\ = {}&\sigma _i\sigma _j x_{i,t}x_{j,t}\rho _{ij}\, \mathrm{d}t \end{aligned}$$

and

2.9 $$\begin{aligned} \mathrm{corr}[\mathrm{d}x_{i,t},\mathrm{d}x_{j,t}|x_{i,t},x_{j,t}]=\rho _{ij}, \end{aligned}$$

where $\mathrm{d}t>0$ and $\mathrm{d}f(t)=f(t+\mathrm{d}t)-f(t)$. That is, $\rho _{ij}$ is the force of linear association between infinitesimal changes of $x_{i,t}$ and $x_{j,t}$. It measures how similar the increasing and decreasing patterns of crime incidence around the expected value are between zip codes. In practice, knowledge of spatial correlations permits reallocating police personnel on certain areas of the city, given an unexpected escalation of criminal activity in a specific district. These areas of police reallocation may not necessarily be adjacent.

It remains the task of inverse parameter estimation for (2.6). We fit the real trend time series $\{s_{1,t}\}_{t\ge 0},\ldots ,\{s_{26,t}\}_{t\ge 0}$ at times $0<1<2<\ldots <120$, by matching $\{s_{1,t}\}_{t\ge 0},\ldots ,\{s_{26,t}\}_{t\ge 0}$ and the proposed processes $\{x_{1,t}\}_{t\ge 0},\ldots ,\{x_{26,t}\}_{t\ge 0}$ given by (2.6), respectively, and calibrating $\mu _1$, $\sigma _1$, $\ldots$, $\mu _{26}$, $\sigma _{26}$ and $\mathrm{A}$. Log-returns $u_{i,j}=\mathrm{ln}(s_{i,j+1})-\mathrm{ln}(s_{i,j})$, $i\in \{1,\ldots ,26\}$, $j\in \{0,1,\ldots ,119\}$, are considered. These are modeled by the random variables

$$\begin{aligned}{} & {} U_{i,j}=\mathrm{ln}(x_{i,j+1})-\mathrm{ln}(x_{i,j})=\left( \mu _i-\frac{1}{2} \sigma _i^2\right) +\sigma _i \Delta B_{i,j},\\{} & {} \quad \Delta B_{i,j}=B_{i,j+1}-B_{i,j}. \end{aligned}$$

By the linear dependence with respect to the Brownian increment $\Delta B_{i,j}$, the distribution of $U_{i,j}$ is normal, with mean value $\mu _i-\frac{1}{2} \sigma _i^2$ and standard deviation $\sigma _i$. By the method of moments, widely used in inferential statistics (Casella and Berger 2002, Section 7.2.1), we estimate $\mu _i$ and $\sigma _i$ as

$$\begin{aligned} {\hat{\mu }}_i-\frac{1}{2} {\hat{\sigma }}_i^2={\overline{u}}_i,\quad {\hat{\sigma }}_i=d_{u_i}, \end{aligned}$$

where ${\overline{u}}_i$ and $d_{u_i}$ are the sample mean and the sample standard deviation of $\{u_{i,0},u_{i,1},\ldots ,u_{i,119}\}$. By isolating, the estimates are

2.10 $$\begin{aligned} {\hat{\mu }}_i={\overline{u}}_i+\frac{1}{2} d_{u_i}^2, \quad {\hat{\sigma }}_i=d_{u_i}. \end{aligned}$$

These values coincide with those in the case of no correlation. Finally, to estimate $\rho _{kl}$, for $k,l\in \{1,\ldots ,26\}$, we notice that the covariance between $U_{k,j}$ and $U_{l,j}$ is $\sigma _k\sigma _l\rho _{kl}$. Therefore, by the method of moments,

2.11 $$\begin{aligned} {\hat{\rho }}_{kl}=\frac{d_{u_k,u_l}}{{\hat{\sigma }}_k{\hat{\sigma }}_l}, \end{aligned}$$

where $d_{u_k,u_l}$ is the sample covariance between $\{u_{k,0},u_{k,1},\ldots ,u_{k,119}\}$ and $\{u_{l,0},u_{l,1},\ldots ,u_{l,119}\}$. When ${\hat{\rho }}_{kl}\ne 0$, we are identifying interaction between the two regions.

As can be seen, our adopted approach does not pose any computational difficulty, regardless of the spatial dimensionality *n*. It allows for fitting past data and forecasting, as well as capturing spatial interactions by correlating the noises. Further details and comparisons with the literature are left for the results and the discussion sections.

## Results

In this section, we present the results obtained when applying the proposed methods on the crime problem. The starting point is the file of 90247 reported crime incidents in Valencia (thefts and robberies in the streets), from January 2010 to December 2020, together with their geographical coordinates. After processing the data adequately, we aim at finding correlations between the zip codes and fitting and forecasting the trend time series, by employing the geometric Brownian motion stochastic process. We use the software Mathematica^®^ (Wolfram Research 2020), version 12.1, installed on an Intel^®^ Core$^{{\mathrm{TM}}}$ i7 CPU 2.9 GHz.

### Data processing

**Fig. 2:**
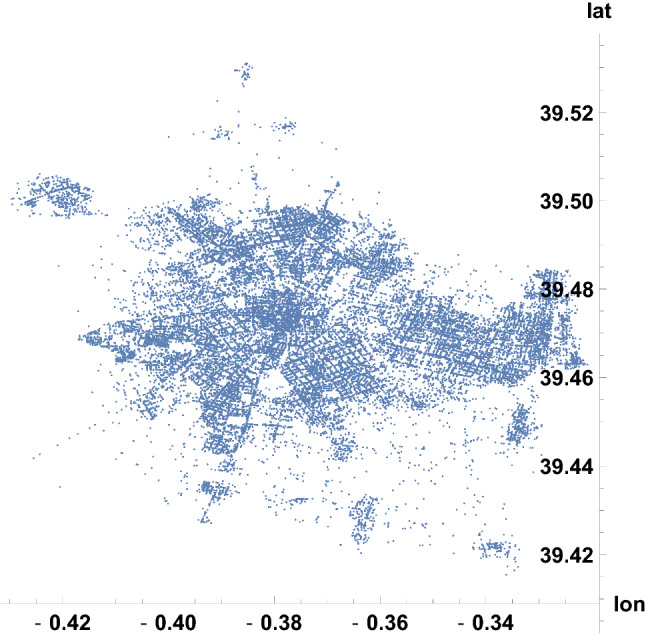
Geographical plot of the 90247 incidents in Valencia communicated to the 112-emergency phone, from January 2010 to December 2020, with their coordinates (longitude, latitude)

**Fig. 3:**
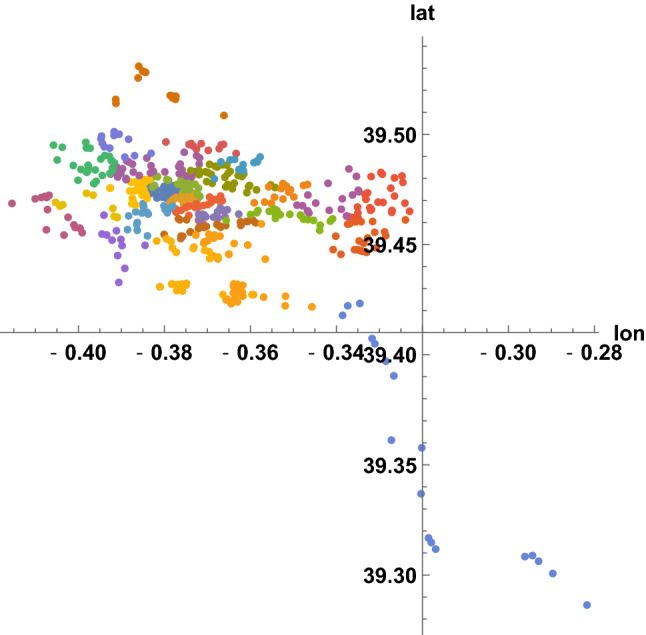
Selected representative positions of the zip codes in Valencia –there are 26 codes in Valencia, from 46001 to 46026, each one with a color–, to assign zip codes to any recorded latitude and longitude of criminal activity by minimizing distances

**Fig. 4:**
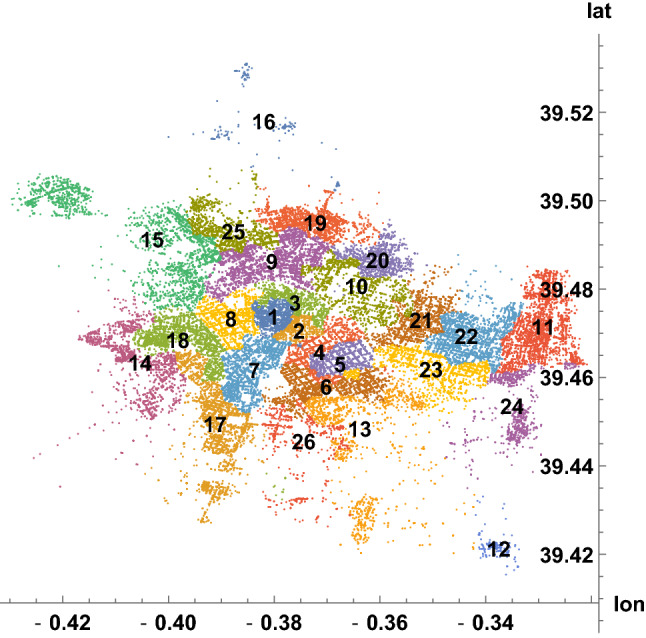
Partition of the 90247 crime incidents in Valencia into 26 patches, period from January 2010 to December 2020. Patch number *i* refers to the zip code $46000+i$. Then, each zip code has a time series of monthly crime counts

Along the eleven years, the crime incidents in Valencia were situated as illustrated in Fig. 2. To analyze spatial-level relations within Valencia, each occurrence is associated to one of the 26 zip codes in the city. As explained in the previous section, we have geographical representatives of the zip codes (around 20 per code), and zip codes are assigned to the 90247 recorded latitudes and longitudes by minimizing distances. In Fig. 3, the representative positions are shown. From them, the partition of the map in Fig. 2 into 26 patches is given in Fig. 4, where label *i* refers to the zip code $46000+i$. This process required around 3 min of CPU time.

### Time-series modeling

**Fig. 5:**
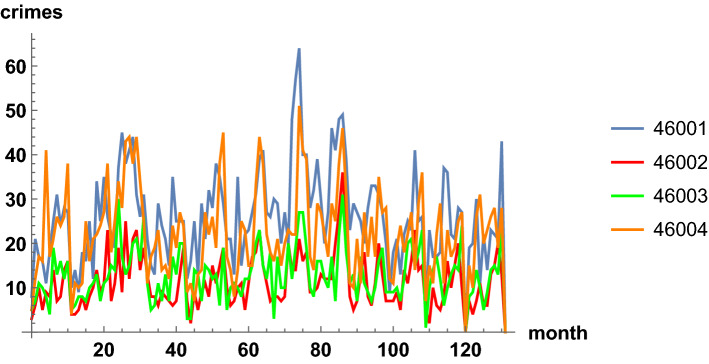
Time series of monthly criminal incidents in Valencia, from January 2010 to December 2020. Four zip codes out of the 26 are shown

**Fig. 6:**
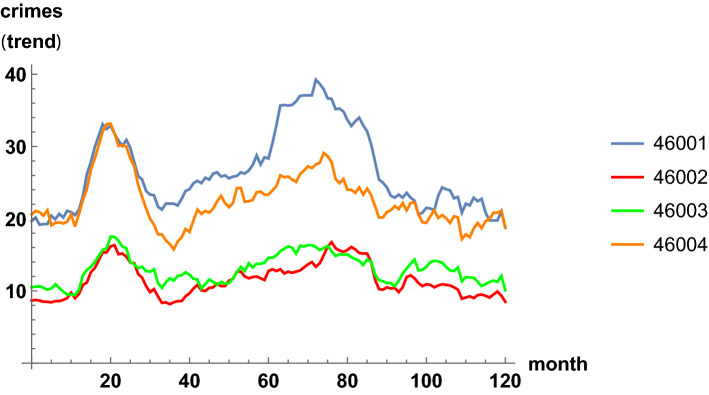
Trend time series of monthly criminal incidents in Valencia from Fig. 5, with annual averages. Four zip codes out of the 26 are shown. The 26 trend time series will be fitted by correlated geometric Brownian processes

The time series with monthly counts for each zip code are the basis of the crime dynamics. According to the previous section, the trend component of each time series is extracted, with annual averages. This smooths out the dynamics, removes seasonal effects, and permits then fitting with a geometric Brownian motion process. In Fig. 5, we plot the time series with monthly counts for the first four zip codes. In Fig. 6, we show the corresponding trend time series. The latter figure is less noisy and it allows for better perceiving patterns and eases the modeling. In the plots, similar increasing and decreasing patterns of incidence are observed between regions, which justifies the analysis of spatial correlations, see (2.8) and (2.9).

The trend time series are modeled by geometric Brownian processes, see the stochastic model (2.6) with solution (2.7). To account for spatial structure, we combine the zip codes and correlate the corresponding Brownian motions. In Table 1, the estimated correlations (2.11) are reported to two significant digits. It gives an idea of the force of association between the crime incidences at district level. The values are naturally positive, since one expects directly proportional relationships. Briefly, the highest correlations are about 0.7, not necessarily for adjacent regions, while near-zero correlations are also present. The information in the table may be of use by practitioners to improve resources on areas of the city, given an unexpected increase of criminal activity in a certain district. Given the processed data, the CPU time to obtain the table was 10 s.

**Table 1:** Criminality correlations between the different zip codes of Valencia

Code	1	2	3	4	5	6	7	8	9	10	11	12	13
1	–												
2	.49	–											
3	.52	.54	–										
4	.50	.61	.55	–									
5	.55	.52	.47	.59	–								
6	.48	.55	.58	.60	.63	–							
7	.41	.45	.50	.52	.57	.57	–						
8	.44	.33	.43	.51	.54	.53	.54	–					
9	.52	.53	.59	.61	.60	.67	.62	.62	–				
10	.52	.49	.54	.56	.50	.41	.49	.51	.53	–			
11	.41	.46	.58	.57	.48	.58	.51	.58	.71	.46	–		
12	.19	.15	.03	.09	.20	.04	.19	.20	.16	.16	.24	–	
13	.44	.35	.39	.57	.32	.42	.44	.53	.55	.46	.58	.08	–
14	.35	.55	.45	.51	.57	.62	.58	.52	.60	.44	.62	.29	.43
15	.42	.45	.49	.51	.51	.52	.63	.59	.55	.41	.63	.20	.54
16	.21	.17	.13	.17	.30	.22	.17	.24	.21	.17	.21	.10	.14
17	.48	.56	.53	.59	.51	.62	.68	.65	.67	.52	.64	.18	.60
18	.60	.48	.56	.57	.58	.54	.61	.64	.65	.61	.65	.21	.54
19	.46	.47	.55	.58	.58	.63	.67	.56	.70	.49	.66	.30	.50
20	.42	.37	.47	.42	.40	.51	.49	.53	.52	.41	.46	.14	.59
21	.52	.44	.55	.60	.53	.53	.60	.48	.60	.54	.49	.09	.50
22	.47	.38	.53	.47	.47	.49	.67	.69	.71	.52	.65	.24	.61
23	.62	.40	.47	.40	.53	.55	.53	.49	.60	.44	.49	.20	.40
24	.48	.38	.36	.51	.42	.45	.43	.43	.45	.38	.44	.32	.44
25	.42	.56	.48	.53	.52	.54	.63	.56	.64	.49	.57	.24	.43
26	.33	.35	.39	.33	.50	.34	.42	.36	.43	.27	.47	.07	.28

Code	14	15	16	17	18	19	20	21	22	23	24	25	26
1													
2													
3													
4													
5													
6													
7													
8													
9													
10													
11													
12													
13													
14	–												
15	.57	–											
16	.23	.13	–										
17	.65	.64	.18	–									
18	.52	.60	.17	.65	–								
19	.62	.66	.14	.70	.66	–							
20	.44	.53	.03	.57	.51	.47	–						
21	.47	.51	.16	.55	.64	.55	.43	–					
22	.64	.62	.16	.67	.69	.64	.59	.58	–				
23	.56	.53	.18	.58	.62	.56	.45	.58	.60	–			
24	.38	.46	.18	.52	.59	.52	.33	.44	.49	.53	–		
25	.63	.54	.22	.69	.60	.64	.37	.54	.59	.54	.44	–	
26	.36	.47	.11	.36	.51	.40	.34	.36	.39	.41	.38	.30	–

Code number *i* refers to the zip code $46000+i$. In the mathematical notation of the paper, we are reporting the estimated values of the parameters $\rho _{ij}$. The upper part of the table is empty because correlations are symmetric. In the diagonal, there are perfect correlations. Given two zip codes, correlation measures the similarity between the patterns of the trend time series around their expected paths, on a linear basis. Higher values of correlation in [0, 1] indicate higher force of this association

**Fig. 7:**
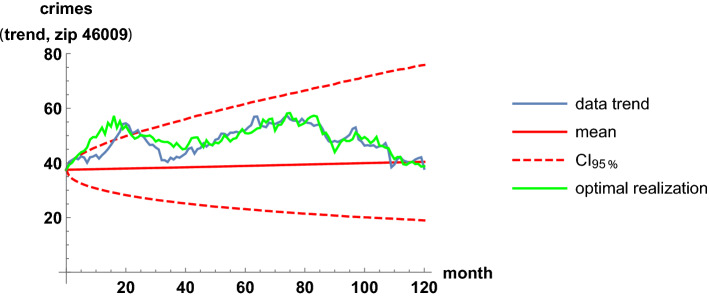
Fit of the trend time series for the zip code 46009. It is based on the mean value, a 0.95 probabilistic region, and an optimal trajectory among $10^5$ realizations

**Fig. 8:**
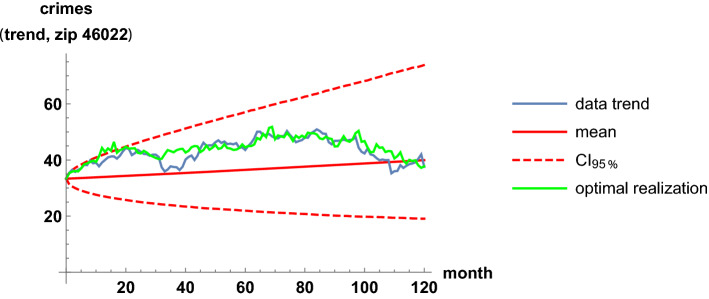
Fit of the trend time series for the zip code 46022. It is based on the mean value, a 0.95 probabilistic region, and an optimal trajectory among $10^5$ realizations

**Fig. 9:**
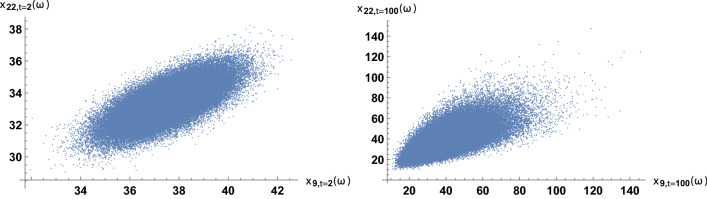
Scatter plots at $t=2$ and $t=100$ by jointly sampling the coupled stochastic model for the zip codes 46009 and 46022. Here, $\omega$ denotes any element of the sample space

We illustrate the fit of pairs of trend time series in the highest-correlation case. The highest correlation occurs for the zip codes 46009 and 46022. Interestingly, the two regions are not geographically close in the map; the code 46009 is in the north, while 46022 is in the east. In Table 2, the estimates of the parameters in the coupled stochastic model are given, after fitting the whole trend series; see (2.10) and (2.11). The computation of this table was instantaneous; only 0.01 s of CPU time were required. The parameters $\mu _9$ and $\mu _{22}$ are global growth rates; these are slightly positive, near zero, because criminality levels are similar at the beginning and at the end of the time span. The parameters $\sigma _9$ and $\sigma _{22}$ are defined as the infinitesimal standard deviations, which account for the random variability. These four parameters are calibrated independently of the correlation coefficient, estimated by 0.71. In Figs. 7 and 8, we show the fit of the trend time series graphically. It is based on the mean value, a 0.95 log-normal probabilistic region, and an optimal trajectory among $10^5$ Euler-Maruyama-type realizations, in the sense of minimizing the sum of the squared differences between the simulated values and the trend data. (We note that the Karhunen-Loève expansion of Brownian motion may also be used for generating trajectories (Lord et al. 2014, Chapter 5). These are usual calculations for model validation (Calatayud et al. 2023a; Cao et al. 2013). Recall that the mean is the curve of the initial deterministic exponential model, which takes into account flows from susceptibility to criminality and vice versa by means of relative risks. The probabilistic interval gathers the trajectories and becomes wider as time passes, by the linear increase of the variance of Brownian motion with time. The fluctuations are mimicked and the time series are accommodated quantitatively. Certainly, the capture of fluctuations would be impossible with deterministic formulations. As the number of runs increases and the ensemble of paths gets larger, it is expected that the least-squares optimal path shows less discrepancy and a better overlap with respect to the trend series. The CPU time for generating Figs. 7 and 8 was around 2 min. Finally, to illustrate the interaction between the two zip codes, we jointly sample their models at $t=2$ and $t=100$ to obtain scatter plots, see Fig. 9.

**Table 2:** Estimates of the parameters when modeling the trends of the zip codes 46009 and 46022 with correlations, by using the method of moments. These two codes are the most-correlated ones

Estimator	Value
${\hat{\mu }}_9$	.00062
${\hat{\mu }}_{22}$	.0015
${\hat{\sigma }}_9$	.032
${\hat{\sigma }}_{22}$	.032
${\hat{\rho }}_{9,22}$	.71

**Fig. 10:**
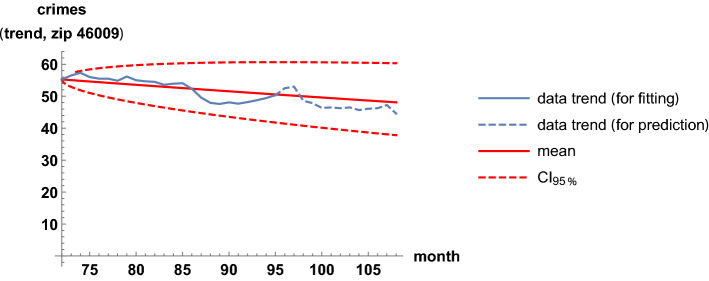
A forecast for the trend time series corresponding to the zip code 46009. It is based on the mean value and a 0.95 probabilistic region. Two years are fixed to calibrate the parameters and then the following year is simulated

An important feature of a model is its capability to predict. To assess it, one fixes a proper training period of the trend time series that is used to calibrate the parameters, and then subsequent times are forecast. The forecast is performed with average values and probabilistic bands, since quantitative pointwise predictions are not possible when working with randomly fluctuating phenomena. Figure 10 illustrates a case of forecast for the zip code 46009, where two years are fixed to calibrate the parameters and then the following year is simulated. Other forecasts are similar, but are not shown here for concision. For real-life applications seeking predictability of crime trends, a short training period with parameter calibrations may be employed to cautiously forecast a few subsequent times.

**Fig. 11:**
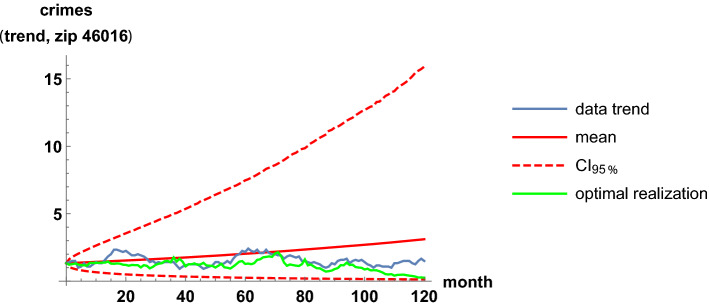
Fit of the trend time series for the zip code 46016. It is based on the mean value, a 0.95 probabilistic region, and an optimal trajectory among $10^5$ realizations

**Fig. 12:**
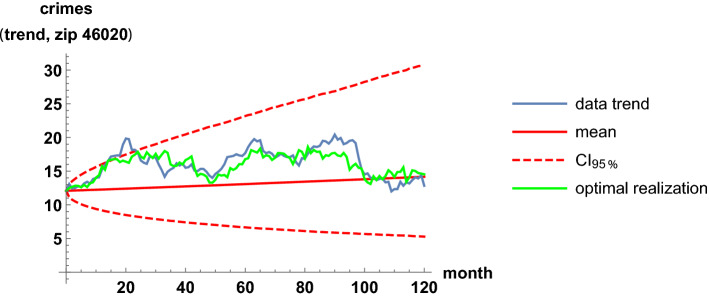
Fit of the trend time series for the zip code 46020. It is based on the mean value, a 0.95 probabilistic region, and an optimal trajectory among $10^5$ realizations

**Fig. 13:**
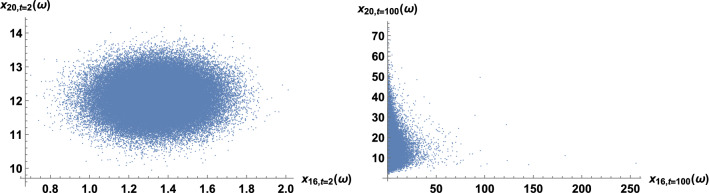
Scatter plots at $t=2$ and $t=100$ by jointly sampling the coupled stochastic model for the zip codes 46016 and 46020. Here, $\omega$ denotes any element of the sample space

**Fig. 14:**
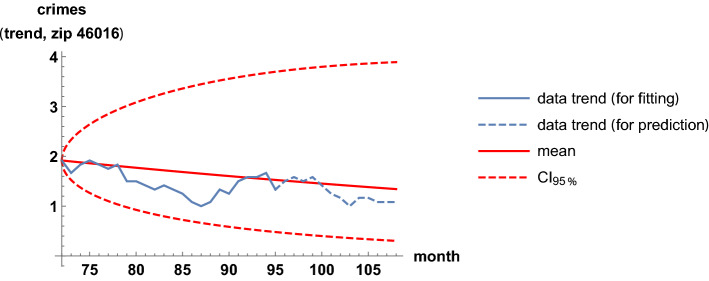
A forecast for the trend time series corresponding to the zip code 46016. It is based on the mean value and a 0.95 probabilistic region. Two years are fixed to calibrate the parameters and then the following year is simulated

Analogous results are shown for the least-correlation case, between the zip codes 46016 and 46020. The two regions are near, separated only by the zip code 46019. Table 3 and Figs. 11, 12, 13 and 14 are analogous to the previous Table 2 and Figs. 7, 8, 9 and 10. It is clearly observed that the trend time series are not related and that the scatter plots do not show any increasing relationship.

**Table 3:** Estimates of the parameters when modeling the trends of the zip codes 46016 and 46020 with correlations, by using the method of moments. These two codes are the least-correlated ones

Estimator	Value
${\hat{\mu }}_{16}$	.0071
${\hat{\mu }}_{20}$	.0013
${\hat{\sigma }}_{16}$	.11
${\hat{\sigma }}_{20}$	.041
${\hat{\rho }}_{16,20}$	.033

## Discussion and conclusions

We segmented the city of Valencia into the 26 zip codes, to partition the *xy*-positions of the 90247 crime data on robberies and thefts. This division gave rise to 26 monthly time series of crime incidence, for a span of eleven years (2010–2020). After an appropriate motivation, the geometric Brownian motion stochastic process was used to model the annual-trend components, where district-level correlations were taken into account. The correlation coefficients corresponded to the pairs of Brownian motions and to the infinitesimal increments of the pairs of response processes. The parameters in the modeling were estimated by the method of moments, with closed-form formulae. The proposed methodology was applied to fit whole trend time series (e.g. Tables 2 and 3, Figs. 7, 8, 9, 11, 12 and 13), obtain the correlations between the zip codes (Table 1), and forecast incidences at short term (e.g. Figs. 10 and 14). In practice, to support law enforcement, one may simulate spatio-temporal situations and identify risky areas and periods from present and past data. This study may be particularly useful for police redistribution, taking into account the existing scarce public resources, and consequently attaining a significant fall of crime (Machin and Marie 2011).

Our approach has several distinctive features. Compared to usual models from partial differential equations or spatial statistics (Cressie and Wikle 2015; Short et al. 2010a, b; Tse and Ward 2015), our paper is not focused on simulating or describing concentrations of crime in particular zones, namely hotspots. We are rather committed to quantitative fitting of real spatio-temporal data and forecasting. On the other hand, compared to usual differential equation models, which include many mechanistic components (social contagion, place attractiveness, etc.) that severely affect the possibility of parameter estimation from real data (Lacey and Tsardakas 2016), our proposal is rather simple both theoretically and computationally. Further, it includes spatial correlations in the formulation, in contrast to similar stochastic models (Calatayud et al. 2023a; Cao et al. 2013). Based on our results, we believe that differential equation-based phenomenological models (Lauer et al. 2021, Section 2.1) shall be considered a tool to assess the evolution of social behaviors. These types of models have certainly been considered in environmental sciences (Calatayud et al. 2022, 2023b; Chowell et al. 2016; Nafidi et al. 2022; Pell et al. 2018) (Zika, Ebola, COVID-19 and CO$_2$ emissions, with certain exponential growths) and turn out to be successful in our context of offenses. Nevertheless, phenomenological forecasting models are limited by the assumption that future incidence will follow the patterns of incidence observed in the past. In any case, this problem may not necessarily be fixed by adding more mechanistic parts (Green and Armstrong 2015), besides complicating estimations and simulations.

The type of stochastic terms incorporated into the model also deserves some further comments. Itô stochastic differential equations were introduced here based on the data fluctuations and dynamics observed in the plots, the need of a probabilistic model for the infinitesimal growth rate, and the facility to later include spatial correlations. Other types of differential-equation randomization have been investigated in the Physics and the environmental literature. In Xiu (2010), Smith (2013), Chen-Charpentier and Stanescu (2010), differential equations with random parameters were studied; in our context, ignoring spatial effects, the corresponding model would be $x_t'=\mu x_t$, where $\mu$ is a time-independent random variable with a probability distribution. However, the solution stochastic process $x_t=x_0\mathrm{e}^{\mu t}$ would not be irregular in such a formulation, but smooth (Neckel and Rupp 2013). This issue could be fixed by incorporating a certain random model error ${\mathcal {E}}_t$, with resulting response process $x_t=x_0\mathrm{e}^{\mu t}+{\mathcal {E}}_t$, and then applying Bayesian or maximum-likelihood inference for parameter estimation (Calatayud and Jornet 2020; Calatayud et al. 2022, 2023b; Smith 2013; Xiu 2010). Nonetheless, a certain structure of the residuals of the deterministic exponential model would then be required, for example, symmetry around zero, homoscedasticity, etc. By inspecting the plots of our paper, that would not be the case. The key to the success of Itô stochastic differential equations is that one starts perturbing the differential $\mathrm{d}x_t$ of the deterministic response, instead of $x_t$ itself.

In the section on *Results*, we included the CPU time of our computations. In our machine, the data processing (partition of the records into the 26 zip codes) required 3 min, the calculation of the correlation table lasted 10 s, the calculation of parameters ($\mu$, $\sigma$ and correlation) for two zip codes along eleven years required 0.01 s, and the simulation of optimal trajectories for two zip codes along eleven years (100,000 realizations) needed around 2 min. It would be interesting to compare these times with other similar methodologies. But, as already commented in the paper, the literature on crime-data fitting with differential equations is very scarce, especially when stochastic effects are considered. In Lacey and Tsardakas (2016); Jane White et al. (2021); Cao et al. (2013), computational costs are not commented. We found a paper on spatio-temporal stochastic differential equations for urban-development modeling (Duan et al. 2009), which proposed a Bayesian hierarchical model with logistic growth and Matérn spatial covariance function; according to the authors, it took about 15 hours to finish the computations. Thus, we think that our ideas may provide a simple and efficient tool to model crime dynamics.

Some modifications and enhancements may be devised from the present study. Four are described next.

First, we used the geometric Brownian motion process in analogy to quantitative finance and stock price evolution. Stock prices are positive, unbounded and do not show mean reversion; our models for crime dynamics assume these properties as well. Alternative formulations are based on Vasicek’s model (which gives rise to the Ornstein-Uhlenbeck process) or the CIR model. These processes possess the properties of mean reversion and long-term finite variance, although the former has positive probability (maybe non-negligible) of getting negative values (Allen 2016). These models are employed in the context of interest rates in finance (Orlando et al. 2020). In criminology, disregarding spatial issues, the use on time series of one or the other models would depend on whether the extent of criminal activities is considered stable and delimited or not asymptotically (for short or moderate periods, this is not specially important), while keeping positivity.

Second, a possible extension of our stochastic differential equation models could be based on the incorporation of jumps. In the financial setting, paper (Synowiec 2008) proposed some jump-diffusion models, by adding a Poisson noise apart from the Gaussian white noise. The proposal stemmed from the fact that log-returns are usually negative asymmetric, leptokurtic and highly fluctuating. In our case, Kolmogorov-Smirnov, Cramér-von Mises and Anderson-Darling tests, based on distances between empirical and hypothesized distribution functions, did not reject normality of the log-returns (acceptance of the null hypothesis at level 0.05 per zip code). Nonetheless, the applicability and goodness of fit of Poisson jumps for criminological time series shall be investigated, besides spatial effects. The most important difficulties would be the construction of the model with correlations and the parameters calibration by maximum likelihood or moments, with a well-posed and convergent optimization procedure.

Third, the growth-rate parameter $\mu$ was assumed to be constant, while perturbing it by means of Gaussian white noise. However, it would be more realistic (albeit more complex) to work with a time-varying parameter, for example, by relating it with certain temporal covariates via link/effect functions (Michelot et al. 2021). These temporal covariates could be based on unemployment rate, economic situation, penal laws, weather, etc. An alternative approach that keeps the parameter constant would be the inclusion of covariates through the noise, viewed as Itô processes themselves; instead of using the differential of Brownian motion at the beginning, one defines a hierarchical model where differentials of covariates are firstly employed (Martínez-Salinas 2020). More research is needed to incorporate these types of mechanisms for fitting spatio-temporal series of crime data.

Fourth, criminality levels at the different zip codes of Valencia were correlated by means of geometric Brownian motions. This methodology gives a spatio-temporal vision on crime evolution. Actually, any time series with fluctuations may be correlated in a similar manner. Currently, we are planning to work not only with crime incidence, but with distances of the incidents to city landmarks (spatial covariates). On a monthly basis, these distances generate other time series. Then, all the time series may be correlated through the stochastic noises. This next study may also be interesting for security policies. Indeed, understanding the relation between changes in crime locations and changes in criminality levels is very important for law enforcement to implement preventive measures.

These topics will be the target of future efforts. Despite the limitations and possible extensions described, we believe that our analysis is a starting point for the use of “financial” stochastic differential equations in mathematical criminology, at the level of spatio-temporal series.

## Data Availability

The data analyzed in this study are available from the authors upon reasonable request.

## Footnotes

1 This intuitive interpretation for the infinitesimal growth rate is only possible under the Itô definition of the stochastic differential equation, which is given by a limit of finite-difference equations in discrete time (Euler-Maruyama discretizations). The Stratonovich interpretation of noise, by contrast, is not appropriate for finite-difference approximations; it is rather used when the interest relies on colored-noise approximations in continuous time (Wong-Zakai theorem) and on the conservation of standard rules of calculus in the stochastic sense. Both interpretations are related operationally. In addition, if $\mu$ is viewed in (2.4) as the average *per capita* growth rate, then the Itô definition corresponds to the arithmetic average, whereas the Stratonovich definition corresponds to the geometric average. More details are given in paper (Braumann 2007).

2 If *f* is a deterministic function, then

$$\begin{aligned} \mathrm{d}f(x_t)=f'(x_t)\mathrm{d}x_t+\frac{1}{2} f''(x_t)(\mathrm{d}x_t)^2, \end{aligned}$$

with $(\mathrm{d}t)^2=0$ and $(\mathrm{d}B_t)^2=\mathrm{d}t\ne 0$. Pick $f=\mathrm{ln}$.
